# Supplementary material for: A Likelihood Approach for Real-Time Calibration of Stochastic Compartmental Epidemic Models
Source: PLoS Comput Biol. 2017 Jan 17;13(1):e1005257. doi: 10.1371/journal.pcbi.1005257 (PMC5240920; doi:10.1371/journal.pcbi.1005257)
Supplement: S1 File — (TAR.GZ) [file pcbi.1005257.s014.tar.gz › HSPH_Online-SI-Revision/MSS10/n100-mild/n100-mild_NEW2_table-4.pdf]

1	newly 3 weeks spec	3503.	3549.81	{2973., 4145.}	10.618	0.046006	newly 3 weeks cumul	8908.	8920.25	{7374., 10 520.}	10.9694	0.0481383	newly 5 weeks spec	3621.	3628.79	{3306., 4022.}	6.4322	0.0278738	newly 5 weeks cumul	16 248.	16 314.	{14 413., 18 525.}	8.34866	0.036389	newly 56 weeks cumul	29 432.	28 232.2	{26 776., 29 844.}	5.15714	0.0230762
2	newly 3 weeks spec	4175.	3659.72	{3146., 4170.}	12.926	0.0620377	newly 3 weeks cumul	10 673.	10 055.7	{8538., 11 363.}	9.56498	0.0441126	newly 5 weeks spec	4247.	3096.53	{2736., 3436.}	27.089	0.138787	newly 5 weeks cumul	19 311.	16 682.3	{14 752., 18 286.}	13.6863	0.0655312	newly 55 weeks cumul	34 724.	25 613.	{24 167., 27 014.}	26.2385	0.13259
3	newly 3 weeks spec	4167.	4713.17	{3674., 5767.}	18.7821	0.0743202	newly 3 weeks cumul	10 718.	11 823.3	{9157., 14 288.}	16.5264	0.0666531	newly 5 weeks spec	4114.	3975.72	{3485., 4394.}	7.12202	0.0323456	newly 5 weeks cumul	19 213.	20 450.2	{16 926., 23 829.}	12.2913	0.050904	newly 58 weeks cumul	31 221.	29 460.6	{27 735., 31 210.}	6.05618	0.0276806
4	newly 3 weeks spec	8072.	8481.44	{5892., 10 061.}	17.6093	0.0860818	newly 3 weeks cumul	18 303.	21 366.2	{10 275., 29 266.}	35.1383	0.155638	newly 5 weeks spec	6729.	3387.69	{1388., 5985.}	51.9297	0.376286	newly 5 weeks cumul	33 292.	30 719.2	{25 371., 34 580.}	10.0502	0.0515041	newly 62 weeks cumul	45 410.	33 795.	{31 740., 35 479.}	25.5782	0.12891
5	newly 3 weeks spec	3934.	4028.78	{3496., 4602.}	8.43925	0.0361785	newly 3 weeks cumul	10 428.	10 578.7	{8988., 11 954.}	8.15084	0.0352194	newly 5 weeks spec	3936.	3604.38	{3186., 3990.}	9.57215	0.0444317	newly 5 weeks cumul	18 434.	18 197.6	{15 947., 20 264.}	6.76267	0.0299441	newly 58 weeks cumul	32 210.	28 124.2	{26 467., 29 570.}	12.7369	0.0596872
6	newly 3 weeks spec	4474.	3951.04	{3386., 4601.}	13.3599	0.0639172	newly 3 weeks cumul	11 885.	10 970.	{9439., 12 759.}	11.2862	0.0524256	newly 5 weeks spec	4394.	3172.65	{2829., 3458.}	27.7959	0.142656	newly 5 weeks cumul	20 970.	17 869.4	{15 791., 20 033.}	15.0489	0.0725364	newly 56 weeks cumul	33 539.	26 233.9	{24 542., 28 031.}	21.7808	0.107231
7	newly 3 weeks spec	5537.	5027.71	{3745., 6134.}	15.7264	0.0758829	newly 3 weeks cumul	11 792.	10 919.6	{8040., 13 620.}	15.8291	0.0756181	newly 5 weeks spec	6137.	5457.59	{4834., 5898.}	13.9444	0.0647285	newly 5 weeks cumul	24 548.	22 176.4	{18 180., 25 307.}	13.0878	0.0622448	newly 61 weeks cumul	35 993.	34 668.7	{32 436., 35 775.}	6.49179	0.0280087
8	newly 3 weeks spec	3810.	3928.75	{3423., 4366.}	8.26378	0.0350677	newly 3 weeks cumul	10 632.	10 870.8	{9365., 12 214.}	8.89917	0.0381502	newly 5 weeks spec	3274.	3190.25	{2819., 3507.}	6.75657	0.0302298	newly 5 weeks cumul	17 804.	17 775.8	{15 819., 19 371.}	6.34251	0.0277801	newly 55 weeks cumul	26 742.	26 261.4	{24 704., 27 391.}	3.58967	0.0159102
9	newly 3 weeks spec	6820.	5565.39	{4653., 6400.}	18.396	0.0911352	newly 3 weeks cumul	16 298.	13 755.4	{11 197., 15 871.}	16.4389	0.0812678	newly 5 weeks spec	6191.	4370.14	{3873., 4774.}	29.4114	0.152564	newly 5 weeks cumul	29 431.	23 477.1	{20 465., 26 157.}	20.2301	0.0998948	newly 60 weeks cumul	43 358.	31 634.8	{30 263., 33 111.}	27.0381	0.137165
10	newly 3 weeks spec	3895.	3038.33	{1883., 3721.}	22.1569	0.123675	newly 3 weeks cumul	10 575.	8929.41	{6449., 10 817.}	17.0673	0.0888487	newly 5 weeks spec	3765.	2389.12	{1156., 2970.}	36.544	0.226284	newly 5 weeks cumul	18 373.	14 121.6	{9071., 16 976.}	23.1466	0.126169	newly 54 weeks cumul	31 484.	21 159.	{11 649., 24 760.}	32.7945	0.189871
11	newly 3 weeks spec	5349.	5154.38	{4057., 6135.}	12.3212	0.0577666	newly 3 weeks cumul	11 903.	11 864.7	{8816., 14 495.}	14.5603	0.066077	newly 5 weeks spec	6127.	4991.47	{4513., 5507.}	18.5332	0.0902368	newly 5 weeks cumul	24 206.	22 389.3	{18 776., 25 513.}	10.1988	0.0488059	newly 58 weeks cumul	41 009.	33 043.2	{31 769., 34 404.}	19.4246	0.0940546
12	newly 3 weeks spec	4227.	3740.04	{3317., 4194.}	11.9957	0.0570609	newly 3 weeks cumul	11 278.	10 707.6	{9610., 11 888.}	8.06925	0.0370273	newly 5 weeks spec	4123.	2909.02	{2569., 3211.}	29.4441	0.153176	newly 5 weeks cumul	19 649.	17 056.	{15 528., 18 365.}	13.3	0.0631779	newly 54 weeks cumul	37 494.	24 645.2	{23 337., 26 036.}	34.269	0.182792
13	newly 3 weeks spec	5374.	5490.23	{4568., 6554.}	11.7691	0.0510151	newly 3 weeks cumul	12 699.	13 088.6	{10 032., 16 104.}	14.2267	0.0614645	newly 5 weeks spec	5146.	4656.18	{4182., 5083.}	10.2246	0.0476604	newly 5 weeks cumul	23 362.	23 252.5	{19 974., 26 471.}	8.50077	0.0376553	newly 59 weeks cumul	36 672.	32 564.9	{31 021., 34 051.}	11.1996	0.0518529
14	newly 3 weeks spec	7541.	6322.21	{5457., 6932.}	18.9851	0.0914999	newly 3 weeks cumul	19 010.	16 642.2	{13 734., 18 921.}	14.4529	0.0696967	newly 5 weeks spec	5669.	4143.35	{3606., 4544.}	30.1028	0.155038	newly 5 weeks cumul	32 236.	26 350.7	{23 131., 28 076.}	20.4454	0.09952	newly 61 weeks cumul	43 111.	32 957.9	{30 504., 33 476.}	25.579	0.127939
15	newly 3 weeks spec	6533.	5646.62	{4555., 6635.}	14.7494	0.073798	newly 3 weeks cumul	14 286.	13 698.5	{10 857., 16 568.}	12.5574	0.059671	newly 5 weeks spec	6860.	4519.5	{4086., 4973.}	34.1181	0.182424	newly 5 weeks cumul	28 288.	23 727.4	{20 100., 26 544.}	16.1537	0.0793495	newly 58 weeks cumul	44 155.	32 393.1	{31 021., 33 773.}	26.6377	0.134724
16	newly 3 weeks spec	2942.	3405.54	{2741., 4004.}	19.8783	0.0792433	newly 3 weeks cumul	7823.	9049.14	{7497., 10 665.}	18.8498	0.0746726	newly 5 weeks spec	2786.	3105.09	{2592., 3654.}	19.8001	0.0865928	newly 5 weeks cumul	13 642.	15 570.8	{12 880., 18 018.}	18.4584	0.0749428	newly 54 weeks cumul	27 181.	25 158.8	{23 269., 27 403.}	7.98087	0.0420111
17	newly 3 weeks spec	6206.	5208.53	{4208., 6223.}	16.6766	0.0839535	newly 3 weeks cumul	13 556.	11 977.9	{9137., 14 899.}	15.9447	0.0787842	newly 5 weeks spec	6327.	4948.38	{4491., 5450.}	21.7895	0.107778	newly 5 weeks cumul	26 549.	22 466.	{19 281., 25 680.}	15.4639	0.0758094	newly 60 weeks cumul	43 173.	32 890.8	{31 345., 34 383.}	23.8163	0.118407
18	newly 3 weeks spec	7370.	14 388.9	{12 041., 16 352.}	98.1661	0.298587	newly 3 weeks cumul	13 588.	26 056.7	{18 705., 32 510.}	95.5988	0.290409	newly 5 weeks spec	8646.	6577.2	{4279., 9179.}	31.2158	0.167237	newly 5 weeks cumul	31 571.	45 008.9	{42 528., 47 625.}	43.3489	0.156186	newly 64 weeks cumul	43 150.	49 222.	{48 046., 50 268.}	14.0717	0.0571087
19	newly 3 weeks spec	4209.	4163.41	{3539., 4978.}	10.5738	0.0466358	newly 3 weeks cumul	10 513.	10 795.4	{9119., 12 438.}	10.358	0.0441604	newly 5 weeks spec	4445.	3671.19	{3189., 4100.}	17.5219	0.0855938	newly 5 weeks cumul	19 470.	18 606.3	{16 169., 21 341.}	8.80026	0.0400916	newly 55 weeks cumul	33 382.	28 596.7	{26 546., 30 464.}	14.3349	0.0678557
20	newly 3 weeks spec	5435.	6595.65	{4834., 7979.}	27.6131	0.101642	newly 3 weeks cumul	9479.	11 425.2	{8352., 13 774.}	26.731	0.0985807	newly 5 weeks spec	7800.	11 326.7	{10 125., 12 333.}	46.7021	0.165552	newly 5 weeks cumul	25 122.	32 776.5	{26 968., 37 273.}	32.3177	0.11942	newly 64 weeks cumul	39 842.	58 193.2	{54 961., 60 251.}	46.4966	0.164768
21	newly 3 weeks spec	6939.	5229.65	{3914., 6126.}	28.4714	0.145844	newly 3 weeks cumul	13 970.	11 091.3	{8407., 13 602.}	24.1467	0.122209	newly 5 weeks spec	7245.	5707.12	{5162., 6167.}	21.6006	0.10677	newly 5 weeks cumul	29 481.	22 843.9	{18 835., 25 275.}	24.8604	0.124592	newly 64 weeks cumul	42 520.	35 366.4	{33 723., 36 140.}	17.6607	0.0842601
22	newly 3 weeks spec	3601.	3087.54	{2656., 3525.}	15.2313	0.0734222	newly 3 weeks cumul	9861.	8737.16	{7589., 9713.}	12.9046	0.0611856	newly 5 weeks spec	3826.	2645.95	{2384., 2959.}	30.8429	0.161721	newly 5 weeks cumul	17 518.	14 345.3	{12 711., 15 751.}	18.1775	0.0889043	newly 54 weeks cumul	36 374.	23 495.4	{22 086., 24 686.}	35.406	0.190274
23	newly 3 weeks spec	5953.	4775.57	{3646., 5922.}	21.0087	0.108445	newly 3 weeks cumul	13 022.	10 777.1	{8151., 13 702.}	20.6932	0.105879	newly 5 weeks spec	6752.	4940.3	{4468., 5434.}	26.832	0.136932	newly 5 weeks cumul	26 990.	20 988.	{17 090., 25 095.}	22.3326	0.114073	newly 59 weeks cumul	42 711.	33 090.6	{31 339., 34 778.}	22.5243	0.111145
24	newly 3 weeks spec	7664.	4931.79	{3618., 6079.}	35.6502	0.201353	newly 3 weeks cumul	15 273.	10 714.5	{7368., 13 308.}	30.0172	0.166055	newly 5 weeks spec	6606.	5414.41	{4826., 5914.}	18.1667	0.0894482	newly 5 weeks cumul	30 012.	21 778.3	{17 735., 25 110.}	27.4626	0.14519	newly 63 weeks cumul	39 935.	34 267.3	{32 645., 35 955.}	14.1924	0.0671289
25	newly 3 weeks spec	4080.	3644.96	{2955., 4252.}	12.9711	0.062669	newly 3 weeks cumul	9690.	9369.53	{7465., 11 132.}	11.157	0.0507917	newly 5 weeks spec	4627.	3453.06	{3046., 3853.}	25.3715	0.128885	newly 5 weeks cumul	18 986.	16 542.	{13 767., 18 928.}	13.3904	0.0649177	newly 54 weeks cumul	37 102.	27 409.4	{25 760., 28 895.}	26.1242	0.131921
26	newly 3 weeks spec	5222.	4954.07	{3914., 5813.}	11.238	0.0541426	newly 3 weeks cumul	12 723.	12 770.1	{9797., 15 154.}	12.6727	0.0567182	newly 5 weeks spec	5352.	3760.6	{2896., 4443.}	29.7347	0.161243	newly 5 weeks cumul	23 692.	21 196.1	{17 454., 24 256.}	11.8515	0.0585283	newly 56 weeks cumul	38 625.	28 405.9	{23 200., 31 056.}	26.4572	0.137044
27	newly 3 weeks spec	4332.	3891.3	{3356., 4460.}	11.7216	0.0553829	newly 3 weeks cumul	10 828.	10 035.2	{8447., 11 588.}	10.4804	0.0488814	newly 5 weeks spec	4581.	3633.94	{3271., 3952.}	20.7356	0.102191	newly 5 weeks cumul	20 103.	17 631.4	{15 815., 19 707.}	12.5963	0.0598886	newly 54 weeks cumul	36 608.	28 192.7	{26 738., 29 683.}	22.9876	0.113852
28	newly 3 weeks spec	3391.	3572.86	{3021., 4078.}	11.3618	0.0480528	newly 3 weeks cumul	9081.	9315.36	{7507., 10 713.}	11.1413	0.0483486	newly 5 weeks spec	3182.	3348.9	{2963., 3648.}	8.62162	0.0360665	newly 5 weeks cumul	15 833.	16 296.9	{13 876., 18 241.}	9.34662	0.0402936	newly 55 weeks cumul	31 926.	26 611.7	{25 250., 28 021.}	16.6457	0.0794721
29	newly 3 weeks spec	3449.	2861.18	{1620., 4108.}	28.0319	0.157605	newly 3 weeks cumul	8407.	7631.26	{4748., 10 169.}	22.4506	0.116022	newly 5 weeks spec	3362.	2565.63	{1148., 3956.}	34.8004	0.214006	newly 5 weeks cumul	15 415.	13 030.1	{7674., 18 704.}	26.2229	0.1441	newly 57 weeks cumul	26 694.	21 024.6	{10 991., 29 733.}	29.3889	0.171061
30	newly 3 weeks spec	6917.	5597.59	{4752., 6426.}	19.1321	0.0961939	newly 3 weeks cumul	15 845.	13 327.1	{10 652., 15 913.}	16.7226	0.0838799	newly 5 weeks spec	6557.	4767.49	{4322., 5171.}	27.2916	0.139259	newly 5 weeks cumul	29 817.	23 703.3	{20 682., 26 431.}	20.5041	0.101738	newly 59 weeks cumul	44 183.	32 904.	{31 622., 34 301.}	25.5279	0.128218
31	newly 3 weeks spec	4050.	3965.57	{3290., 4610.}	10.0141	0.045327	newly 3 weeks cumul	9667.	9959.54	{8580., 11 579.}	10.98	0.0472375	newly 5 weeks spec	4228.	3836.07	{3317., 4322.}	10.6786	0.0500467	newly 5 weeks cumul	18 314.	17 919.7	{15 504., 20 376.}	8.6708	0.0390856	newly 56 weeks cumul	33 046.	29 487.4	{27 542., 31 788.}	10.8371	0.0504044
32	newly 3 weeks spec	3828.	3120.63	{2719., 3559.}	18.9731	0.0935649	newly 3 weeks cumul	10 480.	9075.96	{7864., 9872.}	14.1427	0.0675859	newly 5 weeks spec	3614.	2611.32	{2363., 2876.}	27.7443	0.143868	newly 5 weeks cumul	17 929.	14 641.3	{12 994., 16 212.}	18.51	0.0906588	newly 54 weeks cumul	35 777.	23 173.3	{21 745., 24 593.}	35.2284	0.189961
33	newly 3 weeks spec	6228.	11 495.4	{5185., 15 803.}	93.5687	0.290687	newly 3 weeks cumul	10 354.	18 482.4	{7280., 27 862.}	89.8978	0.282871	newly 5 weeks spec	7272.	10 155.6	{5738., 14 436.}	47.9149	0.162052	newly 5 weeks cumul	26 028.	42 563.4	{34 413., 48 136.}	66.1423	0.221237	newly 64 weeks cumul	32 752.	51 730.5	{48 969., 54 989.}	57.946	0.196706
34	newly 3 weeks spec	7515.	6038.5	{5215., 6732.}	20.4096	0.100857	newly 3 weeks cumul	17 041.	14 956.	{12 304., 17 464.}	14.2452	0.068734	newly 5 weeks spec	6043.	4553.42	{4050., 4940.}	25.95	0.130724	newly 5 weeks cumul	30 687.	25 272.	{22 322., 27 743.}	18.4765	0.0896497	newly 60 weeks cumul	40 483.	33 150.8	{31 506., 33 928.}	19.2461	0.0923851
35	newly 3 weeks spec	11 144.	22 616.3	{20 217., 24 425.}	103.6	0.306675	newly 3 weeks cumul	14 898.	39 469.9	{28 475., 43 800.}	165.767	0.413214	newly 5 weeks spec	7436.	2681.53	{1191., 4908.}	66.8576	0.536289	newly 5 weeks cumul	37 536.	53 030.8	{50 812., 52 807.}	41.2798	0.147978	newly 64 weeks cumul	40 652.	53 659.8	{51 609., 53 347.}	31.998	0.118627
36	newly 3 weeks spec	4855.	14 269.9	{10 528., 16 303.}	194.3	0.460613	newly 3 weeks cumul	9201.	26 317.3	{16 057., 33 807.}	186.948	0.442065	newly 5 weeks spec	5772.	6547.96	{3874., 10 478.}	33.9574	0.12992	newly 5 weeks cumul	20 947.	45 045.8	{40 936., 47 902.}	115.046	0.331441	newly 64 weeks cumul	32 008.	49 152.6	{47 777., 50 233.}	53.5634	0.186181
37	newly 3 weeks spec	5673.	5161.27	{3247., 6494.}	20.8639	0.106436	newly 3 weeks cumul	13 815.	12 643.9	{8500., 14 943.}	14.9851	0.0781935	newly 5 weeks spec	5464.	5376.64	{2556., 8394.}	48.9729	0.234466	newly 5 weeks cumul	25 233.	23 575.2	{14 633., 31 049.}	26.2929	0.128465	newly 57 weeks cumul	39 323.	47 587.5	{21 408., 76 564.}	65.6522	0.259
38	newly 3 weeks spec	6180.	7499.28	{1321., 14 238.}	75.1097	0.390629	newly 3 weeks cumul	10 916.	14 137.5	{2257., 27 519.}	82.8712	0.383839	newly 5 weeks spec	7800.	5683.24	{2091., 10 302.}	45.601	0.318241	newly 5 weeks cumul	26 749.	27 378.2	{6790., 45 065.}	44.6749	0.269787	newly 64 weeks cumul	37 595.	36 697.9	{27 621., 48 962.}	17.5869	0.119769
39	newly 3 weeks spec	5821.	5839.69	{5022., 6643.}	9.6078	0.0423137	newly 3 weeks cumul	14 912.	15 190.7	{12 175., 17 525.}	11.6081	0.050575	newly 5 weeks spec	4560.	4074.57	{3628., 4507.}	11.2151	0.0534068	newly 5 weeks cumul	25 319.	24 554.2	{21 773., 27 107.}	7.44836	0.03382	newly 61 weeks cumul	34 866.	31 577.3	{30 065., 33 130.}	9.43246	0.0435315
40	newly 3 weeks spec	8986.	13 520.5	{12 266., 14 778.}	50.9674	0.177531	newly 3 weeks cumul	18 610.	27 846.4	{22 385., 33 588.}	51.3075	0.176545	newly 5 weeks spec	6981.	6301.21	{3353., 8306.}	21.1885	0.109996	newly 5 weeks cumul	34 664.	45 247.3	{42 414., 47 315.}	30.5312	0.115157	newly 63 weeks cumul	43 420.	50 207.9	{47 080., 52 330.}	15.633	0.0626864
41	newly 3 weeks spec	8035.	5705.46	{3310., 8267.}	30.8692	0.182457	newly 3 weeks cumul	14 755.	10 972.8	{6498., 15 090.}	27.6019	0.157296	newly 5 weeks spec	8819.	7988.64	{5477., 11 462.}	29.7846	0.143657	newly 5 weeks cumul	33 711.	26 611.6	{17 511., 37 691.}	26.7614	0.147004	newly 64 weeks cumul	47 457.	44 536.5	{34 839., 58 840.}	23.2006	0.106894
42	newly 3 weeks spec	6821.	7053.89	{6829., 7280.}	3.53511	0.0148925	newly 3 weeks cumul	14 678.	14 343.	{13 905., 14 695.}	3.22442	0.0141827	newly 5 weeks spec	6589.	10 599.7	{10 364., 10 754.}	60.8695	0.20637	newly 5 weeks cumul	28 856.	34 264.4	{33 522., 34 761.}	18.7427	0.0744663	newly 61 weeks cumul	41 167.	90 165.2	{90 254., 90 613.}	119.023	0.340444
43	newly 3 weeks spec	6531.	6140.73	{3161., 11 719.}	46.0516	0.205188	newly 3 weeks cumul	12 527.	11 817.3	{6322., 20 604.}	39.9341	0.177626	newly 5 weeks spec	8332.	6505.15	{5375., 9141.}	28.4372	0.143821	newly 5 weeks cumul	29 200.	25 526.3	{16 679., 43 645.}	34.3518	0.163165	newly 64 weeks cumul	45 957.	39 094.6	{34 596., 52 196.}	19.7066	0.0950225
44	newly 3 weeks spec	8080.	9279.31	{8083., 10 503.}	15.987	0.0629051	newly 3 weeks cumul	15 523.	17 066.8	{14 776., 19 481.}	12.425	0.0501844	newly 5 weeks spec	8641.	11 979.8	{10 779., 13 436.}	38.6385	0.139858	newly 5 weeks cumul	34 157.	41 328.3	{37 170., 45 985.}	21.0187	0.0813085	newly 63 weeks cumul	46 278.	63 837.6	{58 236., 69 843.}	37.9438	0.137106
45	newly 3 weeks spec	4973.	4029.87	{3373., 4802.}	20.2857	0.101459	newly 3 weeks cumul	11 874.	10 146.1	{8203., 11 931.}	16.9525	0.0831653	newly 5 weeks spec	4999.	3906.98	{3486., 4371.}	21.8448	0.108932	newly 5 weeks cumul	22 191.	18 244.1	{15 710., 21 428.}	18.454	0.0909211	newly 59 weeks cumul	37 756.	29 828.1	{27 891., 32 295.}	20.9978	0.103162
46	newly 3 weeks spec	6137.	5807.06	{5646., 5969.}	5.37624	0.0241119	newly 3 weeks cumul	13 041.	12 013.3	{11 704., 12 281.}	7.88053	0.0357336	newly 5 weeks spec	7235.	9260.07	{9063., 9413.}	27.9899	0.107135	newly 5 weeks cumul	27 607.	29 069.5	{28 513., 29 613.}	5.29757	0.0223687	newly 60 weeks cumul	46 416.	89 940.3	{89 777., 90 076.}	93.77	0.287286
47	newly 3 weeks spec	7497.	9562.43	{5980., 14 206.}	35.2726	0.124684	newly 3 weeks cumul	15 877.	22 362.9	{14 280., 34 470.}	45.9348	0.151135	newly 5 weeks spec	7293.	4365.82	{2598., 5759.}	40.1368	0.238302	newly 5 weeks cumul	31 506.	34 051.9	{24 925., 45 460.}	18.195	0.0730361	newly 60 weeks cumul	45 078.	38 726.1	{33 949., 47 170.}	16.3981	0.0796357
48	newly 3 weeks spec	5435.	4490.93	{3865., 5098.}	17.428	0.0853557	newly 3 weeks cumul	12 559.	11 181.9	{9693., 12 747.}	12.2962	0.0583011	newly 5 weeks spec	5927.	4281.2	{3822., 4647.}	27.7678	0.142684	newly 5 weeks cumul	24 449.	20 124.1	{18 036., 22 416.}	17.6894	0.0860313	newly 55 weeks cumul	39 759.	32 159.	{29 266., 34 390.}	19.1152	0.0929918
49	newly 3 weeks spec	5594.	9196.82	{6748., 10 865.}	67.976	0.22305	newly 3 weeks cumul	12 132.	22 481.5	{10 793., 29 733.}	93.4608	0.282787	newly 5 weeks spec	4978.	3408.95	{1321., 7591.}	50.445	0.31136	newly 5 weeks cumul	22 959.	32 222.2	{25 975., 35 645.}	40.9985	0.146986	newly 61 weeks cumul	32 589.	34 621.2	{32 704., 36 485.}	6.5295	0.0271787
50	newly 3 weeks spec	6652.	13 923.7	{12 639., 15 006.}	109.484	0.319196	newly 3 weeks cumul	14 670.	31 139.6	{23 214., 36 491.}	112.799	0.320881	newly 5 weeks spec	6004.	4114.74	{2609., 5970.}	37.9027	0.214808	newly 5 weeks cumul	27 698.	44 422.2	{41 635., 46 493.}	60.4245	0.20437	newly 62 weeks cumul	37 321.	46 800.1	{45 761., 48 036.}	25.6695	0.0990977
